# Learning Competitive Swarm Optimization

**DOI:** 10.3390/e24020283

**Published:** 2022-02-16

**Authors:** Bożena Borowska

**Affiliations:** Institute of Information Technology, Lodz University of Technology, 93-590 Lodz, Poland; bozena.borowska@p.lodz.pl

**Keywords:** learning particle swarm optimization, competition, competitive swarm, swarm intelligence, optimization

## Abstract

Particle swarm optimization (PSO) is a popular method widely used in solving different optimization problems. Unfortunately, in the case of complex multidimensional problems, PSO encounters some troubles associated with the excessive loss of population diversity and exploration ability. This leads to a deterioration in the effectiveness of the method and premature convergence. In order to prevent these inconveniences, in this paper, a learning competitive swarm optimization algorithm (LCSO) based on the particle swarm optimization method and the competition mechanism is proposed. In the first phase of LCSO, the swarm is divided into sub-swarms, each of which can work in parallel. In each sub-swarm, particles participate in the tournament. The participants of the tournament update their knowledge by learning from their competitors. In the second phase, information is exchanged between sub-swarms. The new algorithm was examined on a set of test functions. To evaluate the effectiveness of the proposed LCSO, the test results were compared with those achieved through the competitive swarm optimizer (CSO), comprehensive particle swarm optimizer (CLPSO), PSO, fully informed particle swarm (FIPS), covariance matrix adaptation evolution strategy (CMA-ES) and heterogeneous comprehensive learning particle swarm optimization (HCLPSO). The experimental results indicate that the proposed approach enhances the entropy of the particle swarm and improves the search process. Moreover, the LCSO algorithm is statistically and significantly more efficient than the other tested methods.

## 1. Introduction

Swarm intelligence (SI) is a branch of artificial intelligence (AI) based on the social behavior of simple organisms occurring in natural environments [1]. The source of its inspiration was observations of the collective behavior of animals such as birds, fishes, bees, bacteria, ants, squirrels and others [2,3,4,5,6,7,8]. There is a number of methods based on swarm intelligence. One of them is particle swarm optimization (PSO).

PSO was developed by Kennedy and Eberhart [9] as a stochastic method of optimization. The standard PSO is based on a swarm of particles, each of which wanders through the search space to find better solutions. The key to the success of the method is the ability to share information found by population individuals. Due to many advantages (simplicity, easy implementation, lack of coding and special operators) [4], the PSO method has been widely applied in solving various optimization problems, including control systems [10], prediction problems [11], image classification [12], energy management [13], bilevel programming problems [14,15], antenna design [16], scheduling problems [17,18], electromagnetism [19,20] and many others.

Unfortunately, in cases of complex, high-dimensional optimization problems with many local optima, PSO can encounter some difficulties associated with preserving a sufficient diversity of particles and maintaining a balance between exploration and exploitation. This leads to premature convergence and renders the results unsatisfactory. In order to avoid these inconveniences, various improved versions of the PSO have been developed. The improvements rely on [21]:

- Adjustment of parameters. Shi and Eberhart [22] indicated that the performance of the PSO method depends primarily on the inertia weight. In their opinion, the best results give inertial weight, which decreases linearly from 0.9 to 0.4. Zhang et al. [23] and Niu et al. [24] proposed to use a random inertia weight. In contrast to Zhang et al. [23] and Niu et. al. [24], Clerc [25] suggested that all coefficients should rather be constant and proved that inertia weight w = 0.729 and factors c1 = c2 = 1.494 can increase the rate of convergence. Venter and Sobieszczański [26] found that the PSO method is more effective when the acceleration coefficients are constant but different. According to them, the social coefficient should be greater than the cognitive one, and they proved that c2 = 2.5 and c1 = 1.5 produce superior performance. Nonlinear, dynamically changing coefficients were proposed by Borowska [27]. In this approach, the values of coefficients were affected by the performance of PSO and the number of iterations. In order to more efficiently control the searching process, Ratnaweera et al. [28] recommended time-varying coefficients (TVACs). An approach based on fuzzy systems was proposed by Chen [29].
- Topology. Topological structure has a great influence on the performance of the PSO algorithm. According to Kennedy and Mendes [30], a proper topology significantly improves the exploration ability of PSO. Lin et al. [31] and Borowska [32] indicated that the ring topology can help maintain swarm diversity and improve the algorithm’s adaptability. An approach based on multi-swarm structure was proposed by Chen et al. [33] and Niu [24]. In turn, a two-layer cascading structure was recommended by Gong et al. [34]. To alleviate premature convergence, Mendes et al. [35] introduced a fully informed swarm in which particles are updated based on the best locations of their neighbors. A PSO variant with adaptive time-varying topology connectivity (PSO-ATVTC) was developed by Lim et al. [36]. Carvalho et al. [37] proposed a particle topology based on the clan structure. The dynamic Clan PSO topology was described by Bastos-Filho et al. [38]. Shen et al. [39] proposed a multi-stage search strategy supplemented by mutual repulsion and attraction among particles. The proposed algorithm increases the entropy of the particle population and leads to a more balanced search process.
- Combining PSO with other methods. In order to obtain higher-quality solutions and enhance the performance of PSO, in many papers, researchers merge two or more different methods or their advantageous elements. Ali et al. [40] and Sharma et al. [41] combined PSO with genetic operators. A modified particle swarm optimization algorithm with simulated annealing strategy (SA) was proposed by Shieh et al. [42]. A PSO method with ant colony optimization (ACO) was developed by Holden et al. [43]. Cooperation of many swarms and four other methods for improving the efficiency of PSO was applied by Liu and Zhou [44]. Not only did the authors combine multi-population-based particle swarm optimization with the simulated annealing method but also with co-evolution theory, quantum behavior theory and mutation strategy. A different approach was presented by Cheng et al. [45]. To improve the exploration ability of PSO, they used a multi-swarm framework combining the feedback mechanism with the convergence strategy and the mutation strategy. The proposed approach helps reach a balance between exploration and exploitation and reduces the risk of premature convergence.
- Adaptation of learning strategy. This approach allows particles to acquire knowledge from high-quality exemplars. In order to increase the adaptability of PSO, Ye et al. [46] developed dynamic learning strategy. A comprehensive learning strategy based on historical knowledge about particle position was recommended by Liang et al. [47] and was also developed by Lin et al. [48]. Instead of individual learning of particles based on their historical knowledge, Cheng and Jin [49] introduced a social learning mechanism using sorting of swarm and learning from demonstrators (any better particles) of the current swarm. The method turned out to be effective and computationally efficient. A learning strategy with operators of the genetic algorithm (GA) and a modified updating equation based on exemplars was proposed by Gong et al. [34]. To enhance diversity and improve the efficiency of PSO, Lin et al. [31] merged PSO with genetic operators and also connected them with global learning strategy and ring topology. Learning strategy with genetic operators and interlaced ring topology was also proposed by Borowska [32]. To improve the searching process, Niu et al. [50] recommended applying learning multi-swarm PSO based on a symbiosis.

In order to improve the performance of the particle swarm optimization method, Cheng et al. [21] introduced a competitive swarm optimizer (CSO) based on PSO. In CSO, neither the personal best position of particles nor the global best position is required. Instead of them, a simple pairwise competition mechanism within one single swarm was introduced. Particles do not need knowledge about their historical positions as they learn only from the winner.

Unfortunately, although the CSO method has better search capability than traditional PSO, it does not always perform well and obtain expected results for complex optimization problems. Difficulties are associated with the loss of population diversity too quickly and maintaining a balance between exploration and exploitation. This leads to a deterioration in the effectiveness of the method and premature convergence.

To reduce these inconveniences, ensure diversity of particles and limit the risk of getting stuck in the local optimum, in this paper, a new learning competitive swarm optimization called LCSO is presented. The proposed approach is based on the particle swarm optimization method (PSO) and a competition concept. In LCSO, particles do not use information about their personal best position and global best particle in the swarm; instead of that, the competition mechanism was applied but in a different way than in CSO. In LCSO, the swarm is divided into sub-swarms, each of which can work independently. In each sub-swarm, three particles participate in the tournament. The participants who lost the tournament learn from their competitors. The winners take part in the tournament between sub-swarms. The new algorithm was examined on a set of test functions. To evaluate the effectiveness of the proposed LCSO, the test results were compared with those achieved through the competitive swarm optimizer (CSO) [21], comprehensive particle swarm optimizer (CLPSO) [47], PSO [51], fully informed particle swarm (FIPS) [35], the covariance matrix adaptation evolution strategy (CMA-ES) [52] and heterogeneous comprehensive learning particle swarm optimization (HCLPSO) [53].

## 2. The PSO Method

As mentioned in the introduction, particle swarm optimization is a method based on swarm intelligence and the collective behavior of animal societies. It was first proposed by Kennedy and Eberhart [9] as a new simple optimization tool. Because of its effectiveness, it has been regarded as a powerful method of optimization and has become a competitive approach to the genetic algorithm and other artificial intelligence tools [1].

In PSO, the optimization process is performed by the population of individuals called a swarm of particles. The swarm consists of *N* particles that move in the D-dimensional search space. The individual particle within the swarm can be considered as a point of the search space determined by two vectors: *X_i_* = (*x_i_*_1_*, x_i_*_2_*, …, x_iD_*) named a position vector and *V_i_* = (*v_i_*_1_*, v_i_*_2_*, …, v_iD_*) named a velocity vector. Both vectors are randomly generated at the first step of the PSO algorithm. Each particle roams through the search space according to its velocity vector *V_i_* and remembers its personal best position *pbest_i_* = (*pbest_i_*_1_*, pbest_i_*_2_*, …, pbest_iD_*) that it found during its way and the best position *gbest* = (*gbest*_1_*, gbest*_2_*, …, gbest_D_*) found in an entire swarm. The particles are also evaluated on their quality, which is measured based on the objective function of the optimized task. The particle wandering in the search space is performed according to the velocity equation determined as follows:

(1) $$V_{i}\left(t+1\right)=w\cdot V_{i}\left(t\right)+c_{1}\cdot r_{1}\left(pbest_{i}-X_{i}\left(t\right)\right)+c_{2}\cdot r_{2}\left(gbest-X_{i}\left(t\right)\right)$$

The change of the particle position is realized by adding the velocity vector to its position vector according to the given equation:

(2) $$X_{i}\left(t+1\right)=X_{i}\left(t\right)+V_{i}\left(t+1\right)$$

where *t* is the iteration number, *w* means the inertia weight (that determines the impact of the previous velocity of the particle on its current velocity), factors *c*_1_ and *c*_2_ are acceleration coefficients, and *r*_1_ and *r*_2_ represent two random numbers uniformly distributed between 0 and 1. The pseudo code of the standard PSO method is presented in Algorithm 1.
**Algorithm 1** Pseudo code of the PSO algorithm.   Determine the size of the swarm    **for**
*j* = 1 to size of the swarm **do**     Generate an initial position and velocity of the particle,     Evaluate the particle;     Determine the *pbest* (personal best position) of the particle    **end**
    Select the *gbest* (the best position) found in the swarm    **while** termination criterion is not met **do**     **for**
*j* = 1 to size of the swarm **do**      Update the velocity and position of each particle according to Equations (1) and (2)      Evaluate new position of the particle;      Update the *pbest* (personal best position) of each particle     **end**
     Update the *gbest* (the best position) found in the swarm    **end**


## 3. The Proposed LCSO Algorithm

The proposed LCSO (learning competitive swarm optimization) is a two-stage-learning-based method, which combines particle swarm optimization with a competition concept. In the first stage, particles take part in the tournaments within sub-swarms. In the second stage, tournaments are held between sub-swarms. Although LCSO is based on PSO, the knowledge acquisition mechanism is different. In LCSO, particles do not store any historical information about both their personal best position and global best particle in the swarm. Instead, particles derive knowledge from their better competitors.

In the first stage of LCSO, the swarm of *N* particles is divided into *p* sub-swarms. In every iteration, each particle within a sub-swarm participates in a tournament. In the sub-swarms, the tournaments are organized independently. In one tournament, participate 3 randomly selected particles. In one iteration, a particle takes part in a competition only once. The best particle of the tournament (winner) goes to the next iteration without updating. The particle that has finished the tournament as a second one (runner-up) learns from the winner according to the equations:

(3) $$V_{s}\left(t+1\right)\;=r_{1}\cdot V_{s}\left(t\right)\;+\;r_{2}\cdot \left(X_{w}\left(t\right)-X_{s}\left(t\right)\right)$$

(4) $$X_{s}\left(t+1\right)\;=X_{s}\left(t\right)\;+\;V_{s}\left(t+1\right)$$

The particle that took the last place (loser) learns from its competitors according to the following formulas:

(5) $$V_{l}\left(t+1\right)\;=r_{1}\cdot V_{l}\left(t\right)\;+\;r_{2}\left(X_{w}\left(t\right)-X_{l}\left(t\right)\right)\;+\;r_{3}\left(X_{s}\left(t\right)-X_{l}\left(t\right)\right)$$

(6) $$X_{l}\left(t+1\right)\;=X_{l}\left(t\right)\;+\;V_{l}\left(t+1\right)$$

where *X_w_* is the position of the best particle (winner), *V_s_* and *X_s_* are velocity and position of the particle that got second place (runner-up), *V_l_* and *X_l_* are velocity and position of the particle that got last place (loser), *t* is the iteration number, and *r*_1_, *r*_2_ and *r*_3_ are randomly generated numbers in the range [0, 1].

**Figure 1:**
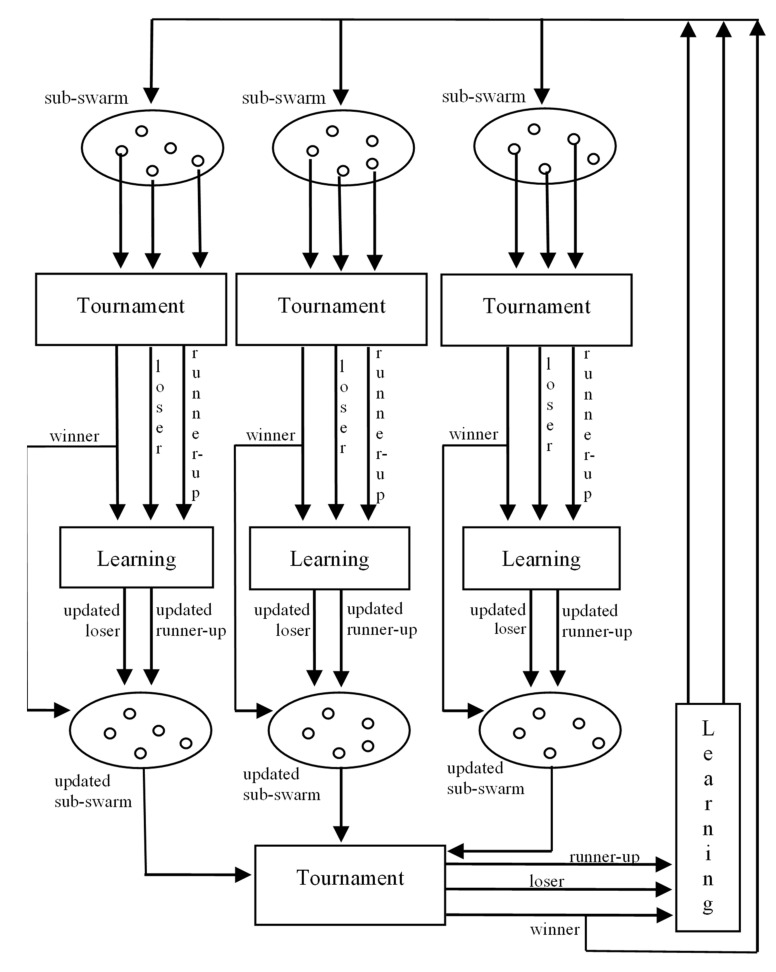
Learning LCSO with 3 sub-populations.

This means that for the sub-swarm of *M* particles, only two out of three particles are updated, whereas one in three particles of each sub-swarm go to the next stage without updating. Then the tournaments are organized between sub-swarms. For each sub-swarm, one particle from the set of the winners is selected. Subsequently, the (each three) particles participate in the tournament. The particle that finished the tournament as a second one (runner-up) learns from the winner according to Equations (3) and (4). The particle that got last place (loser) learns from its competitors according to (5) and (6). Next, the tournaments are organized again in sub-swarms. Figure 1 illustrates the concept of learning LCSO.

This means that among particles that participate in competition between sub-swarms only two out of three particles are updated and one-third of them go to the next iteration without updating.

The proposed sub-swarms topology limits the excessive loss of swarm diversity and helps maintain a balance between exploration and exploitation. The competition strategy (in the sub-swarm) promotes the interaction among particles of the sub-swarm. The introduced learning strategy in the sub-swarm ensures the exchange of information between them. The tournament strategy among the winners ensures sharing of information between sub-swarms. In this way, the sub-swarm that falls into the local optimum has a chance to jump out of it by learning from the winners of other sub-swarms.

The pseudo code of the LCSO method is summarized in Algorithm 2.
**Algorithm 2** Pseudo code of the LCSO algorithm. *FEs* is the number of fitness evaluations. The termination condition is the maximum number of fitness evaluations (*maxFEs*).  Determine the size of the swarm;  Determine the number of sub-swarms;  Determine number of particles in the sub-swarms;  Randomly initialize position and velocity of the particles in sub-swarms;  *t* = 0;  **while** termination criterion (*FEs ≤ maxFEs*) is not met **do**   Evaluate the particles fitness;   Parallel in each sub-swarm organize tournament independently:    **while** sub-swarm ≠ Ø **do**     Randomly select three particles, compare their fitness and determine winner *X_w_*, runner-up *X_r_* and loser *X_l_*     Update runner-up’s position according to (3) and (4)     Update loser’s position according to (5) and (6)    **end**
   Organize tournament between sub-swarms:   Randomly select one particle from the winners of each sub-swarm    **while** set of selected winners ≠ Ø **do**     Randomly select three particles     compare fitness of the selected particles to determine winner *X_w_*, runner-up *X_r_* and loser *X_l_*     Update runner-up’s position according to (3) and (4)     Update loser’s position according to (5) and (6)    **end**
   *t* = *t* + 1;  **end**
  output the best solution

## 4. Results

The tests of the proposed algorithm were performed on a set of benchmark functions, sixteen of which are presented in this article and described in Table 1.

The first five test functions (*f*_1_–*f*_5_) are unimodal, the next six (*f*_6_–*f*_11_) are multimodal, whereas the remaining ones *(f*_12_–*f*_16_) are rotated multimodal functions. The performance of the presented LCSO algorithm for all functions was compared with the results using the competitive swarm optimizer (CSO) [21], comprehensive particle swarm optimizer (CLPSO) [47], particle swarm optimization (PSO) [51], fully informed particle swarm (FIPS) [35], covariance matrix adaptation evolution strategy (CMA-ES) [52] and heterogeneous comprehensive learning particle swarm optimization (HCLPSO) [53].

The experiments were conducted on a PC with an Intel Core i7-3632QM 2.2 GHz CPU and Microsoft Windows 10 64-bit operating system. The LCSO was implemented in C with Microsoft Visual Studio 2010 Enterprise.

The parameter settings of the comparison algorithms are listed in Table 2. The details of algorithms used for comparison can be found in [21,35,47,51,53].

For all tested functions, the experiments with dimension size *D* = 30 were conducted. The population size of all algorithms is *N* = 72, whereas the maximum number of evaluations is 10,000. The number of sub-swarms *p* = 3. The exemplary results of the tests are summarized in Table 3, and the presented values were averaged over 32 runs. The best results are shown in bold.

**Figure 2:**
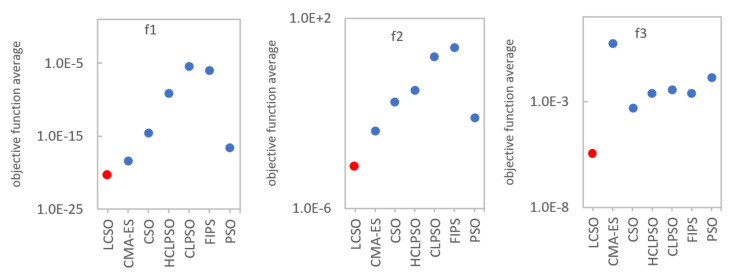
Average value of the objective function (*f*_1_–*f*_3_) for the indicated algorithm.

**Figure 3:**
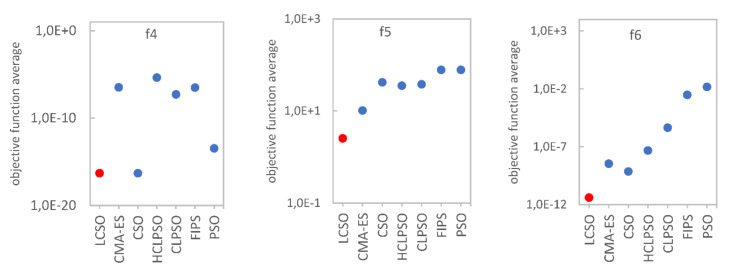

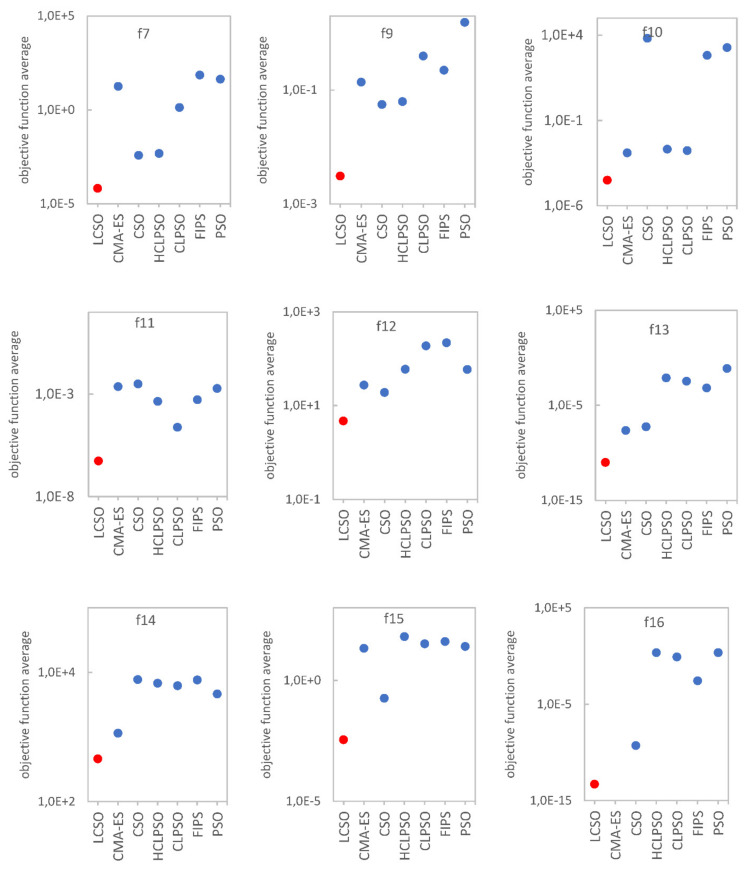
Average value of the objective function (*f*_4_–*f*_16_) for the indicated algorithm.

**Figure 4:**
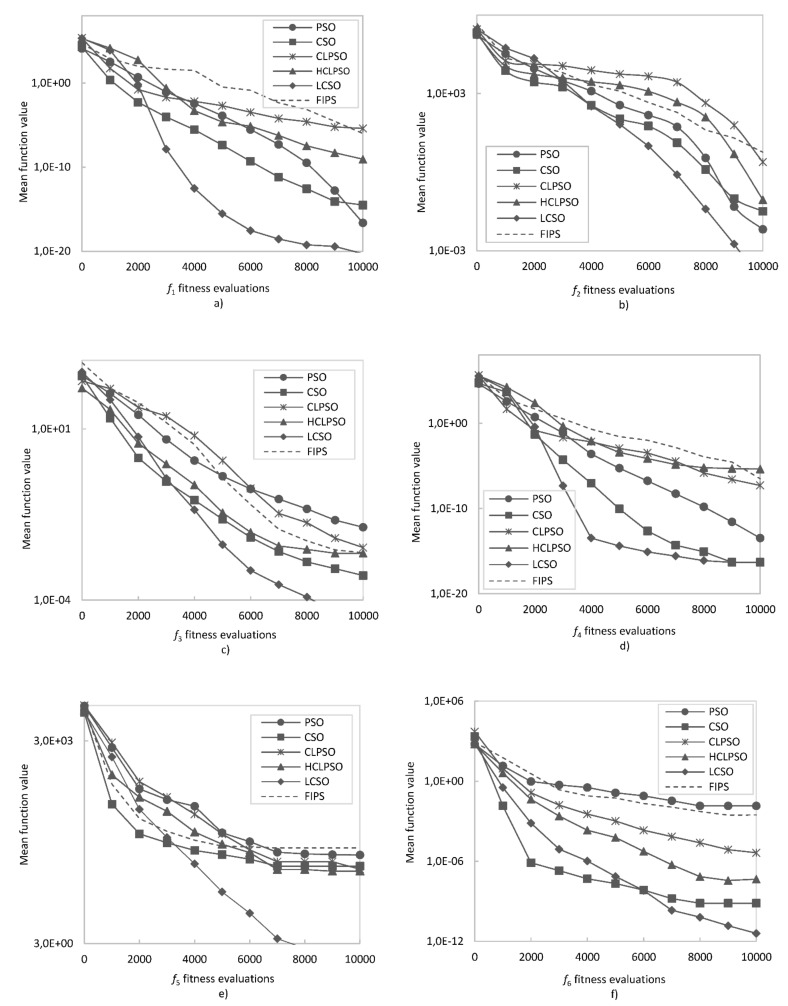
Convergence curve for: (**a**) *f*_1_ function; (**b**) *f*_2_ function; (**c**) *f*_3_ function; (**d**) *f*_4_ function; (**e**) *f*_5_ function; (**f**) *f*_6_ function.

**Figure 5:**
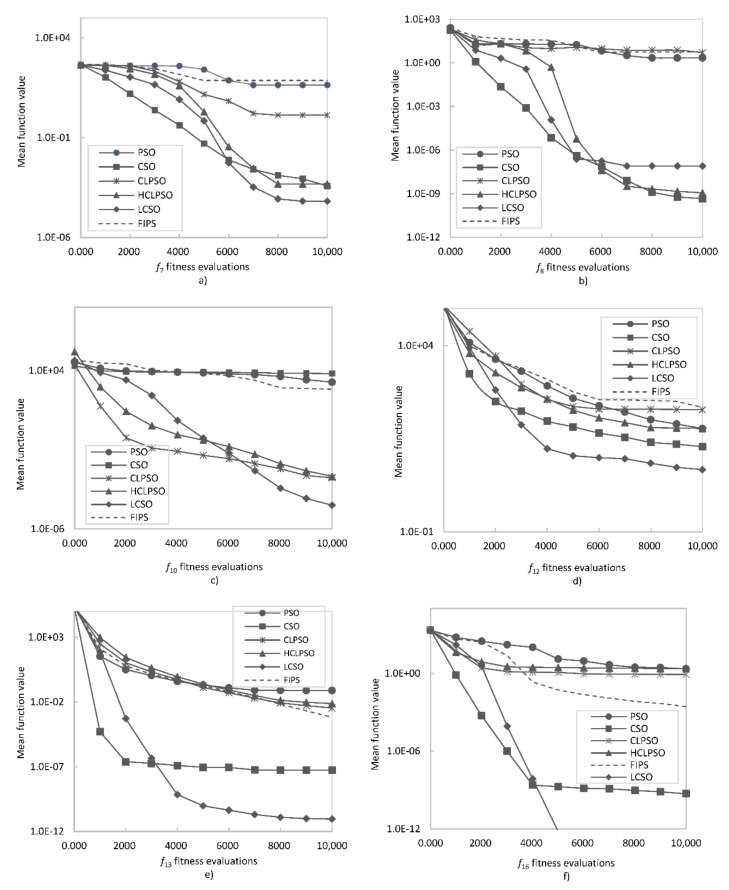
Convergence curve for: (**a**) *f*_7_ function, (**b**) *f*_8_ function, (**c**) *f*_10_ function, (**d**) *f*_12_ function, (**e**) *f*_13_ function), (**f**) *f*_16_ function.

To facilitate understanding of the results, the comparison of the algorithm’s effectiveness on a logarithmic scale is shown in Figure 2 and Figure 3. In order to compare the convergence rate of the tested algorithms, the convergence curves on six representative functions were plotted and presented in Figure 4 and Figure 5. To evaluate the effectiveness of the proposed method, a statistical *t*-test was conducted. For all comparisons, a confidence level of 0.05 was used. The *t*-values between LCSO and the other considered algorithms for 30 dimensions are presented in Table 4.

The results of the tests indicate that the LCSO method with the proposed approach is effective as it achieved superior performance over the other tested algorithms.

For the unimodal functions, LCSO obtained the best results (although the standard deviation values were not always the lowest) among all the algorithms, and only for *f*_4_, the results of LCSO and CSO were comparable, but LCSO turned out to be more stable. The weakest results for the *f*_1_ function were obtained by CLPSO, for *f*_2_ and *f*_3_ by FIPS and for *f*_5_ by PSO. For multimodal functions, in the case of *f*_6_, *f*_9_, *f*_10_, *f*_11_, *f*_12_, *f*_14_, *f*_15_ and *f*_16_, the LCSO method also achieved the best outcomes and was more stable than the other tested algorithms. In the case of *f*_7_ and *f*_13_ functions, LCSO achieved higher performance compared to the other methods, but it was not as stable as them. In the case of *f*_7_, the HCLPSO method turned out to be more stable than the others. For *f*_13_ function, the CSO method was more stable than LCSO. In the case of *f*_8_ function, LCSO performed worse than CSO and HCLPSO but much better than CLPSO, PSO and FIPS. In the case of *f*_16_ function, the results gained by CSO are also much better than those achieved by CLPSO, PSO, FIPS and even HCLPSO but not as good as LCSO. In all the tests, the least effective was PSO as it obtained the poorest results. Particles of PSO moved irregularly and had a tendency to fall and stop in the local minima. The convergence curves presented in Figure 4 and Figure 5 indicate that, for unimodal functions, LCSO converged slower in the early stage than most of the compared methods. At this stage, CSO was found to perform with the highest rate. However, after about 3000 iterations, LCSO surpasses CSO and other tested methods. Regarding multimodal functions, at an early stage, LCSO also converges slower than CSO but faster than CLPSO, PSO, FIPS and HCLPSO. In the middle stage, LCSO surpasses CSO and converges faster than the other algorithms (except *f*_8_), but it needs as many as 6000 iterations to achieve this. However, for *f*_8_ function, LCSO slows down in the middle stage and ultimately converges slower than CSO and HCLPSO.

The obtained results confirm that the proposed method is effective and efficient. The advantages of the proposed LCSO method include:

- The use of sub-swarms helps maintain the diversity of the population and keep the balance between the global exploration and local exploitation;
- Particles learning from the winners can effectively search space for a better position;
- Good information found by sub-swarm is not lost;
- Particles can learn from the useful information found by other sub-swarms;
- In each iteration, the position and velocity of only two out of three particles is updated which significantly reduces the cost of computations;
- Particles do not need to remember their personal best position; instead, the competition mechanism is applied;
- LCSO can obtain better results and convergence than the other algorithms.

Based on the *t*-test results summarized in Table 4, it can be concluded that the performance of the proposed learning competitive swarm optimization (LCSO) algorithm is significantly better than the other methods with a 95% confidence level in a statistically meaningful way.

In order to assess the effectiveness of the LCSO method for a larger dimension of the search space, functions with dimensions of 100, 500 and 1000 were investigated. The tests were performed with a population of *N* = 99 particles. The maximum number of evaluations was 80,000. The results of the tests were averaged over 10 runs and are presented in Table 5.

It should be noted that, despite the increase of the search space dimensions, the proposed LCSO method is still effective for functions *f*_1_, *f*_6_, *f*_7_, *f*_8_, *f*_9_, *f*_12_, *f*_13_ and *f*_16_. An increase in the number of dimensions requires an increase in the size of the population. It also entails an increase in computation costs.

## 5. Conclusions

In this paper, a new learning competitive swarm optimization algorithm (LCSO) based on the particle swarm optimization method and competition mechanism was proposed. In LCSO, particles do not have to remember their personal best positions; instead of that, they participate in the tournament. The tournaments take place in sub-swarms as well as between them. The participants of the tournament update their positions by learning from their competitors.

The efficiency of the LCSO method was tested on a set of benchmark functions. Then the test results were compared with four different variants of PSO, including CSO, CLPSO, PSO, CMA-ES, FIPS and HCLPSO. The obtained results indicate that the proposed LCSO is faster and more effective than the other examined algorithms. The competition mechanism used in LCSO helps maintain particle diversity and improve its exploration ability.

Future work will focus on the implementation of the LCSO algorithm in real-world optimization problems. The application of the LCSO method in machine learning and image compression is also a promising area of research.

## Figures and Tables

**Table 1:** Test functions.

Function	Formula	*F*_min_	Range
Sphere	$f_{1}=\overset{n}{\underset{i=1}{\sum }}x_{i}{}^{2}$	0	[−100, 100]*^n^*
Schwefel 1.2	$f_{2}=\overset{n}{\underset{i=1}{\sum }}(\overset{i}{\underset{j=1}{\sum }}x_{j}{)}^{2}$	0	[−100, 100]*^n^*
Quartic	$f_{3}=\overset{n}{\underset{i=1}{\sum }}ix_{i}^{4}+random\left[0,1\right)$	0	[−100, 100]*^n^*
Schwefel 2.22	$f_{4}=\overset{n}{\underset{i=1}{\sum }}|x_{i}|+\overset{n}{\underset{i=1}{\prod }}\left|x_{i}\right|$	0	[−10; 10]*^n^*
Rosenbrock	$f_{5}=\overset{n-1}{\underset{i=1}{\sum }}[100{\left(x_{i+1}-x_{i}^{2}\right)}^{2}+{\left(x_{i}-1\right)}^{2}$	0	[−30, 30]*^n^*
Griewank	$f_{6}=\frac{1}{4000}\overset{n}{\underset{i=1}{\sum }}x_{i}^{2}-\overset{n}{\underset{i=1}{\prod }}\cos\left(\frac{x_{i}}{\sqrt{i}}\right)+1$	0	[−600, 600]*^n^*
Rastrigin	$f_{7}=\overset{n}{\underset{i=1}{\sum }}\left(x_{i}^{2}-10\cos\left(2\pi x_{i}\right)+10\right)$	0	[−5.12, 5.12]*^n^*
Ackley	$f_{8}=-20\;\exp\left(-0.2\sqrt{\frac{1}{n}\overset{n}{\underset{i=1}{\sum }}x_{i}^{2}}\right)$ $-\exp\left(\frac{1}{n}\overset{n}{\underset{i=1}{\sum }}\cos(2\pi *x_{i})\right)+20+e$	0	[−32, 32]*^n^*
Zakharov	$f_{9}=-\overset{n}{\underset{i=1}{\sum }}x_{i}+(\overset{n}{\underset{i=1}{\sum }}\frac{i}{2}x_{i}{)}^{2}+(\overset{n}{\underset{i=1}{\sum }}\frac{i}{2}x_{i}{)}^{4}$	0	[−10, 10]*^n^*
Schwefel	$f_{10}=418.9829\;\cdot \;n-\overset{n}{\underset{i=1}{\sum }}(x_{i}\cdot \sin(|x_{i}{|}^{0.5})$	0	[−400, 400]*^n^*
Weierstrass	$f_{11}=\overset{n}{\underset{i=1}{\sum }}\left(\overset{kmax}{\underset{k=0}{\sum }}[a^{k}\cos\left(2\pi b^{k}\left(x_{i}+0.5\right)\right)]\right)$ $\;-n\overset{kmax}{\underset{k=0}{\sum }}[a^{k}\cos\left(2\pi b^{k}\cdot 0.5\right)]$ *a* = 0.5, *b* = 3, *k*_max_ = 20	0	[−0.5, 0.5]*^n^*
Rotated Rastrigin	$f_{12}=\overset{n}{\underset{i=1}{\sum }}\left(z_{i}^{2}-10\cos\left(2\pi z_{i}\right)+10\right),$ z=x · M	0	[−5.12, 5.12]*^n^*
Rotated Griewank	$f_{13}=\frac{1}{4000}\overset{n}{\underset{i=1}{\sum }}z_{i}^{2}-\overset{n}{\underset{i=1}{\prod }}\cos\left(\frac{z_{i}}{\sqrt{i}}\right)+1,\;$ z=x · M	0	[−600, 600]^n^
Rotated Schwefel	$f_{14}=418.9829\;\cdot \;n-\overset{n}{\underset{i=1}{\sum }}y_{i}$ $y_{i}=\left\{\begin{array}{c} x_{i}\sin({\left|x_{i}\right|}^{0.5}\;\;\;\;\;\;\;\;\;\;\;\;\;\;if\left|x_{i}\right|\leq 500\\ 0.001{\left(\left|x_{i}\right|-500\right)}^{2}\;if\left|x_{i}\right|>500 \end{array}\right.$ $for\;i=1,2\ldots .n,\;\;\;\;x=x^{\prime }+420.96\;\;$ $\;x^{\prime }=M\left(z-420.96\right)$	0	[−400, 400]*^n^*
Rotated Weierstrass	$f_{15}=\overset{n}{\underset{i=1}{\sum }}\left(\overset{kmax}{\underset{k=0}{\sum }}[a^{k}\cos\left(2\pi b^{k}\left(x_{i}+0.5\right)\right)]\right)$ $\;-n\overset{kmax}{\underset{k=0}{\sum }}[a^{k}\cos\left(2\pi b^{k}\cdot 0.5\right)]$ *a* = 0.5, *b* = 3, *k*_max_ = 20, y=M · x	0	[−0.5, 0.5]*^n^*
Rotated Ackley	$\;\;f_{16}=-20\;\exp\left(-0.2\sqrt{\frac{1}{n}\overset{n}{\underset{i=1}{\sum }}x_{i}^{2}}\right)$ $-\exp\left(\frac{1}{n}\overset{n}{\underset{i=1}{\sum }}\cos(2\pi *x_{i})\right)+20+e$ z=x · M	0	[−32, 32]*^n^*

**Table 2:** Parameter settings of algorithms.

Algorithm	Inertia Weight *w*	Acceleration Coefficients *c*_1_, *c*_2_, *c*	Other Parameters
CLPSO	*w* = 0.9–0.4	*c* = 1.496	-
FIPS		*c* = 2.05	χ = 0.729
PSO	*w* = 0.9–0.4	*c*_1_ = 2.0, *c*_2_ = 2.0	-
HCLPSO	*w* = 0.99–0.2	*c*_1_ = 2.5–0.5, *c*_2_ = 0.5–2.5, *c* = 3–1.5	-
CSO	*w* = 0.7298	*c* = 1.49618	*p*_m_ = 0.01, s_g_ = 7

**Table 3:** Comparison results.

Function		CLPSO	PSO	HCLPSO	CSO	CMA-ES	FIPS	LCSO
*f*_1_	Mean	4.15E-06	2.43E-17	8.93E-10	3.22E-15	4.16E-19	1.03E-06	**5.24E-21**
	Std	3.81E-06	3.15E-17	7.11E-10	3.75E-15	4.07E-19	1.59E-06	2.19E-21
*f*_2_	Mean	2.43E-01	6.54E-03	8.93E-02	3.22E-02	1.78E-03	4.88E-00	**5.63E-05**
	Std	3.15E-01	4.81E-03	7.11E-02	3.75E-02	4.02E-04	5.37E-00	4.39E-05
*f*_3_	Mean	3.42E-03	1.34E-02	2.33E-03	5.22E-04	5.21E-01	2.41E+03	**3.61E-06**
	Std	5.17E-03	9.06E-03	7.94E-04	5.08E-04	2.79E-03	1.47E+03	6.42E-06
*f*_4_	Mean	5.16E-08	3.44E-14	4.33E-06	**4.88E-17**	3.25E-07	3.17E-07	**4.86E-17**
	Std	1.04E-07	2.80E-14	1.13E-05	4.63E-16	2.81E-07	4.54E-07	**3.40E-16**
*f*_5_	Mean	3.78E+01	6.14E+01	3.53E+01	4.20E+01	1.02E+01	2.82E+01	**2.54E+00**
	Std	1.86E+01	5.80E+01	5.14E+00	3.15E+01	1.61E+00	2.14E+01	5.72E+00
*f*_6_	Mean	4.43E-06	1.45E-02	4.61E-08	7.21E-10	3.34E-09	2.93E-03	**3.98E-12**
	Std	1.01E-05	1.29E-02	5.42E-08	5.36E-10	1.02E-10	4.18E-03	3.41E-11
*f*_7_	Mean	1.34E+00	4.38E+01	4.78E-04	3.80E-04	1.8E+01	7.32E+01	**6.54E-06**
	Std	6.16E-01	8.75E+00	7.05E-05	2.52E-04	7.35E-01	2.25E+01	3.77E-05
*f*_8_	Mean	5.12E-01	2.24E+00	1.15E-09	**4.60E-10**	7.35E-12	5.74E+00	8.12E-08
	Std	8.39E-01	1.46E+00	3.67E-10	1.88E-09	1.14E-12	6.18E-01	7.93E-09
*f*_9_	Mean	3.97E-01	1.56E+00	6.30E-02	5.61E-02	1.39E-01	2.25E-01	**3.08E-03**
	Std	3.12E-01	1.25E+00	5.47E-02	3.95E-02	2.61E-02	2.43E-01	2.95E-03
*f*_10_	Mean	1.71E-03	1.86E+03	2.08E-03	6.53E+03	1.25E-03	6.62E+02	**3.15E-05**
	Std	1.59E-03	2.07E+02	1.37E-03	3.87E+03	1.94E-03	4.94E+02	4.39E-05
*f*_11_	Mean	2.39E-05	1.89E-03	4.38E-04	3.15E-03	2.32E-03	5.29E-04	**5.37E-07**
	Std	2.12E-05	1.67E-03	3.65E-04	1.97E-03	1.85E-03	1.92E-04	3.71E-07
*f*_12_	Mean	1.89E+02	5.94E+01	5.98E+01	1.92E+01	2.74E+01	2.21E+02	**7.67E+00**
	Std	5.14E+01	6.73E+00	4.26E+01	5.07E+00	1.30E-01	2.80E+01	4.02E+00
*f*_13_	Mean	3.44E-03	7.68E-02	7.53E-03	5.41E-09	2.28E-08	6.67E-04	**9.16E-12**
	Std	4.96E-03	8.39E-02	8.66E-03	7.92E-09	3.46E-09	1.02E-03	7.85E-11
*f*_14_	Mean	6.23E+03	4.66E+03	6.83E+03	7.78E+03	1.15E+03	7.65E+03	**4.59E+02**
	Std	4.18E+03	3.19E+03	3.99E+03	3.54E+03	3.29E+03	3.61E+03	6.34E+02
*f*_15_	Mean	3.27E+01	2.55E+01	6.45E+01	1.83E-01	2.12E+01	4.07E+01	**3.47E-03**
	Std	3.39E+01	3.12E+01	4.73E+01	2.78E-01	1.96E+00	8.65E+00	2.51E-03
*f*_16_	Mean	8.27E-01	2.25E+00	2.31E+00	5.11E-10	1.76E+00	2.71E-03	**5.09E-14**
	Std	6.54E-02	6.58E-01	5.15E-02	5.16E-10	5.04E-01	2.03E-03	6.71E-14

**Table 4:** The *t*-test results.

Function	*t*-Value between CLPSO and LCSO	*t*-Value between PSO and LCSO	*t*-Value between HCLPSO and LCSO	*t*-Value between CSO and LCSO	*t*-Value between FIPS and LCSO	Two-Tailed *p* between CLPSO and LCSO	Two-Tailed *p* between PSO and LCSO	Two-Tailed *p* between HCLPSO and LCSO	Two-Tailed *p* between CSO and LCSO	Two-Tailed *p* between FIPS and LCSO
*f*_1_	6.16E+00	4.36E+00	7.10E+00	4.86E+00	3.66E+00	1.60E-08	3.18E-05	1.95E-10	4.51E-06	4.02E-04
*f*_2_	4.36E+00	7.62E+00	7.10E+00	4.85E+00	5.14E+00	3.18E-05	1.59E-11	1.99E-10	4.67E-06	1.40E-06
*f*_3_	3.74E+00	8.36E+00	1.66E+01	5.77E+00	9.27E+00	3.12E-04	4.21E-13	3.50E-30	9.19E-08	4.55E-15
*f*_4_	2.82E+00	6.94E+00	2.16E+00	N/A	3.95E+00	5.89E-03	4.28E-10	3.30E-02	N/A	1.47E-04
*f*_5_	1.02E+01	5.71E+00	2.41E+01	6.97E+00	6.55E+00	3.47E-17	1.19E-07	6.31E-43	3.67E-10	2.64E-09
*f*_6_	2.49E+00	6.36E+00	4.81E+00	7.55E+00	3.97E+00	1.45E-02	6.49E-09	5.44E-06	2.26E-11	1.40E-04
*f*_7_	1.23E+01	2.83E+01	6.95E+00	4.66E+00	1.84E+01	1.35E-21	5.57E-49	4.01E-10	1.00E-05	1.45E-33
*f*_8_	3.45E+00	8.68E+00	N/A	N/A	5.25E+01	8.23E-04	8.84E-14	N/A	N/A	1.35E-73
*f*_9_	7.14E+00	7.04E+00	6.19E+00	7.57E+00	5.17E+00	1.63E-10	2.60E-10	1.43E-08	2.05E-11	1.26E-06
*f*_10_	5.97E+00	5.08E+01	8.45E+00	9.55E+00	7.58E+00	3.81E-08	3.10E-72	2.70E-13	1.18E-15	1.97E-11
*f*_11_	6.23E+00	6.40E+00	6.78E+00	9.04E+00	1.56E+01	1.15E-08	5.35E-09	9.11E-10	1.44E-14	3.01E-28
*f*_12_	1.93E+01	3.61E+01	6.67E+00	9.76E+00	4.13E+01	4.12E-35	1.84E-58	1.51E-09	4.01E-16	8.48E-64
*f*_13_	3.80E+00	5.01E+00	4.76E+00	3.75E+00	3.58E+00	2.52E-04	2.38E-06	6.62E-06	3.00E-04	5.39E-04
*f*_14_	7.48E+00	7.07E+00	8.64E+00	1.11E+01	1.07E+01	3.26E-11	2.25E-10	1.09E-13	3.95E-19	2.93E-18
*f*_15_	5.28E+00	4.48E+00	7.47E+00	3.54E+00	2.58E+01	7.69E-07	2.06E-05	3.39E-11	6.20E-04	1.94E-45
*f*_16_	6.93E+01	1.87E+01	2.46E+02	5.42E+00	7.31E+00	4.80E-85	3.78E-34	1.53E-138	4.21E-07	7.22E-11

**Table 5:** Results on 100, 500 and 1000 dimensional functions.

Function	*D* = 100	*D* = 500	*D* = 1000
*f*_1_	1.97E-23	2.03E-11	2.14E-09
*f*_2_	4.62E-01	7.39E+01	2.17E+02
*f*_3_	1.95E-01	1.38E+04	2.01E+05
*f*_4_	5.16E-02	8.41E+00	3.56E+02
*f*_5_	9.38E+01	5.22E+02	9.72E+02
*f*_6_	1.02E-18	5.46E-12	3.65E-10
*f*_7_	1.06E-13	3.71E-08	5.29E-06
*f*_8_	5.12E-14	2.24E-05	4.15E-03
*f*_9_	7.06E-07	4.67E-04	5.07E-02
*f*_10_	3.18E+04	1.82E+05	3.94E+05
*f*_11_	1.37E-02	2.56E+01	4.61E+03
*f*_12_	1.25E-12	3.77E-09	6.29E-08
*f*_13_	1.03E-14	6.32E-09	5.07E-08
*f*_14_	2.56E+04	1.69E+05	3.83E+05
*f*_15_	1.42E+01	3.70E+02	7.26E+02
*f*_16_	2.07E-13	3.24E-07	4.19E-06

## Data Availability

Not applicable.
